# Vesicular Stomatitis Virus-Based Vaccine Protects Mice against Crimean-Congo Hemorrhagic Fever

**DOI:** 10.1038/s41598-019-44210-6

**Published:** 2019-05-23

**Authors:** Sergio E. Rodriguez, Robert W. Cross, Karla A. Fenton, Dennis A. Bente, Chad E. Mire, Thomas W. Geisbert

**Affiliations:** 1 Galveston National Laboratory, University of Texas Medical Branch, Galveston, TX 77555 USA; 2 Department of Microbiology & Immunology, University of Texas Medical Branch, Galveston, TX 77555 USA

**Keywords:** Vaccines, Virology, Vaccines, Virology

## Abstract

Crimean-Congo hemorrhagic fever virus (CCHFV), a tick-borne bunyavirus, can cause a life-threatening hemorrhagic syndrome in humans but not in its animal host. The virus is widely distributed throughout southeastern Europe, the Middle East, Africa, and Asia. Disease management has proven difficult and there are no broadly licensed vaccines or therapeutics. Recombinant vesicular stomatitis viruses (rVSV) expressing foreign glycoproteins (GP) have shown promise as experimental vaccines for several viral hemorrhagic fevers. Here, we developed and assessed a replication competent rVSV vector expressing the CCHFV glycoprotein precursor (GPC), which encodes CCHFV structural glycoproteins. This construct drives strong expression of CCHFV-GP, *in vitro*. Using these vectors, we vaccinated STAT-1 knock-out mice, an animal model for CCHFV. The vector was tolerated and 100% efficacious against challenge from a clinical strain of CCHFV. Anti-CCHFV-GP IgG and neutralizing antibody titers were observed in surviving animals. This study demonstrates that a rVSV expressing only the CCHFV-GP has the potential to serve as a replication competent vaccine platform against CCHF infections.

## Introduction

Crimean-Congo hemorrhagic fever virus (CCHFV) is a highly pathogenic zoonotic agent in the *Orthonairovirus* genus within *Nairoviridae*^1^. The principal reservoirs for CCHFV are ixodid hard ticks primarily belonging to the genus *Hyalomma*^2^. These ticks maintain the virus in nature by feeding on small mammals, ungulates, and birds within thirty countries across the Eastern hemisphere^3^. Human infection can occur from the bite of an infected tick, exposure to infected animal products, or through nosocomial transmission^4^. CCHFV case-fatality rates in most outbreaks range from 3–30%, though higher rates have been documented in some instances^3,5^. CCHFV is categorized as a Category A priority pathogen by the US National Institutes of Health due to its associated morbidity and mortality, potential for public health/societal impact, as well as a lack of approved therapeutic options or US/EU licensed vaccines for treatment. The guanosine analogue ribavirin has been suggested as a therapeutic when given early in human infections; however, efficacy has not been clearly demonstrated in clinical trials for CCHF^6^. For these reasons, vaccines and therapeutic countermeasures against CCHFV are currently under development.

Full or partial mature CCHFV particles contain single stranded, tri-partite, negative sense RNA genomes with small (S), medium (M), and large (L) segments, respectively encoding the structural nucleoprotein (NP), two envelope proteins (G_N_ and G_C_) and the viral RNA-dependent-RNA-polymerase (RdRp)^3^. The M-segment contains a 5.1 kilobase open-reading frame which codes for a glycoprotein precursor polypeptide (GPC)^7^. Host cell processing, cleavage events, and post-translational modifications of this GPC yield the two mature structural glycoproteins G_N_ and G_C_, along with several non-structural glycoproteins which aid in structural G_N_ and G_C_ maturation^7–12^. The two glycoproteins are likely responsible for pertinent events in the viral replication cycle such as viral attachment, cell entry, tissue tropism(s), and induction of protective immune response as seen similarly with other members of *Bunyavirales*^13,14^. The latter has been demonstrated for CCHFV using monoclonal antibodies (MAb) directed against G_N_ and G_C_, which have demonstrated *in vitro* neutralization in tissue culture and *in vivo* passive protection in suckling mice^13–15^. These data suggest that the GPC would be an important antiviral target for therapeutic and vaccine efforts.

Currently, there are several experimental vaccine candidates that have relied on the GPC as an antigenic component, which have been evaluated in immunocompromised signal transducer and activator of transcription 1 knock-out (STAT-1^−/−^), interferon α/β receptor knock-out (IFNAR), or interferon receptor antibody transiently suppressed (IS) mouse models for CCHFV, as they recapitulate clinical illness and are uniformly lethal models for CCHF^16–20^. Vaccine candidate approaches have focused on either DNA expression of CCHFV antigens in host tissues, replication deficient viral-like particles, inactivated whole virus preparations, subunit antigen preparations, or vectored vaccinia virus vaccines^16,20–25^. Two of these preparations, a prime and boost strategy using modified recombinant Vaccinia virus (strain: Ankara) [MVA] encoding the GPC, and a prime, boost, and boost strategy with a DNA based vaccine encoding separate NP, G_N_, and G_C_ antigens, have provided promising results with up to 100% protection in the IFNAR mouse model for CCHFV^23,25^. Although CCHFV-NP by itself as an antigen in the MVA vaccine platform has failed to provide protection^26^.

Recombinant vesicular stomatitis viruses (rVSV) have been developed and evaluated as promising experimental vaccines for several pathogens, often requiring only a single-dose to induce protection^27–31^. The rVSV platform has been experimentally evaluated for both durability and safety^32–34^, and two rVSV vaccines, one for human immunodeficiency virus (HIV)^35^ and a second for *Zaire ebolavirus* (EBOV), have been tested in human clinical trials^36–38^. For these reasons, we hypothesized that rVSV vectors expressing CCHFV-GPC could elicit a protective response in a lethal animal model for CCHF. The aim of our study was to design, generate, characterize, and evaluate a rVSV vector encoding the CCHFV-GPC as an experimental vaccine for CCHFV.

## Results

**Figure 1:**
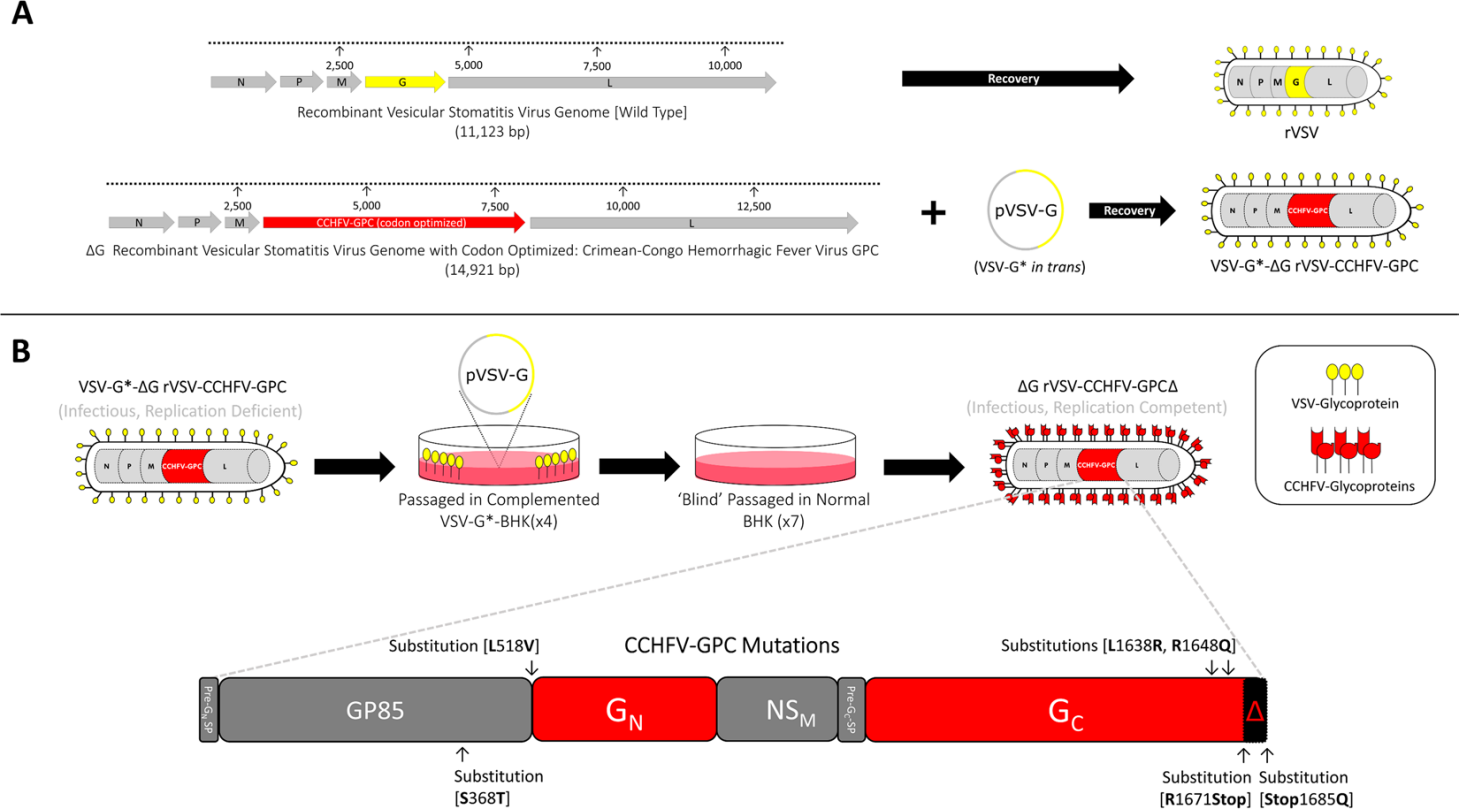
rVSV vector designs and vaccine study strategy. (**A**) Generating a replication deficient vaccine vector: Genome organization comparing VSV (wild-type) genome (in grey arrows) and the rVSV vector expressing the CCHFV-GPC codon optimized open reading frame. N; nucleoprotein, P; phosphoprotein, M; matrix protein, and L; large polymerase protein. The rVSV vector had the VSV glycoprotein open reading frame (yellow arrow) exchanged with the open reading frame coding for the full glycoprotein precursor gene (GPC) of CCHFV (red arrow). Using a T7 driven DNA clone recovery system, a complemented VSV-G* recombinant was generated containing the CCHFV-GPC open reading frame (VSV-G*-ΔGrVSV-CCHFV-GPC). (**B**) Generating a replication competent vaccine vector: VSV-G*-ΔGrVSV-CCHFV-GPC is infectious due to the VSV-G* complementation; however, VSV-G is needed *in trans* to effectively replicate in cell culture. Multiple passages of this vector through VSV-G* complemented and un-complemented BHK cells resulted in a replication competent vaccine vector (ΔGrVSV-CCHFV-GPCΔ). Next generation sequencing revealed six nonsynonymous mutations in the open reading frame of the CCHFV-GPC. These mutations resulted in the truncation of fourteen amino acids off the end of GPC C-terminal tail of the glycoprotein (G_C_).

Our initial attempts using the DNA clone recovery system designed by Lawson *et al*., failed to produce infectious ΔGrVSV with CCHFV-GPC (Fig. 1A)^39^. To overcome this, we used a modification based on a system described by Whitt^40^ relying on *in trans* VSV glycoprotein (G) complementation (VSV-G*) of ΔGrVSV virions. This technique allowed for VSV-G, incorporation into recoveries to facilitate efficient assembly of the rVSVΔG-CCHFV-GPC genome without the need for CCHFV-GPC to participate in initial infection of recovered virions (Fig. 1A). We recovered a virion containing the CCHFV-GPC in the genome with VSV-G complementation (designated VSV-G*-ΔGrVSV-CCHFV-GPC), which contributed to a single-cycle infection; unless VSV-G is provided *in trans* this virus will not replicate effectively in cell culture.

After the initial recovery of VSV-G*-ΔGrVSV-CCHFV-GPC, this virus was passaged on VSV-G complemented BHK cells and passaged onto un-complemented (‘normal’) BHK cells. We were unable to isolate infectious virus from initial supernatants, however, seven total serial passages of supernatants on un-complemented BHK cells resulted in eventual cytopathic effect (CPE) in cell culture with foci/plaque formations appearing on the monolayers. These monolayers with CPE were harvested for RNA and were stained for CCHFV-GPC antigens via immunofluorescence assay and found to be positive (data not shown). This replication competent construct was designated ΔGrVSV-CCHFV-GPCΔ (Fig. 1B). Sanger sequencing of both constructs, using primers for the VSV backbone and CCHFV-GPC ORF, was carried out which confirmed a rVSVΔG-CCHFV-GPC genome and revealed several single nucleotide polymorphisms (SNP) (data not shown). Next generation sequencing (NGS) was then performed to confirm Sanger results and further detail the SNPs within the entire genomes of both constructs (Supplemental Table 1). NGS sequencing demonstrated fourteen identical single nucleotide polymorphisms (SNP) preserved between the replication deficient and replication competent constructs; with the replication competent construct possessing four additional non-synonymous mutations within the CCHFV-GPC (Fig. 1B, Supplemental Table 1); henceforth referred to as GPCΔ. Two of these mutations within the CCHFV-GPC of the replication competent construct, resulted in the truncation of fourteen amino acids off the C-terminal tail of the G_C_ (Fig. 1B).

**Figure 2:**
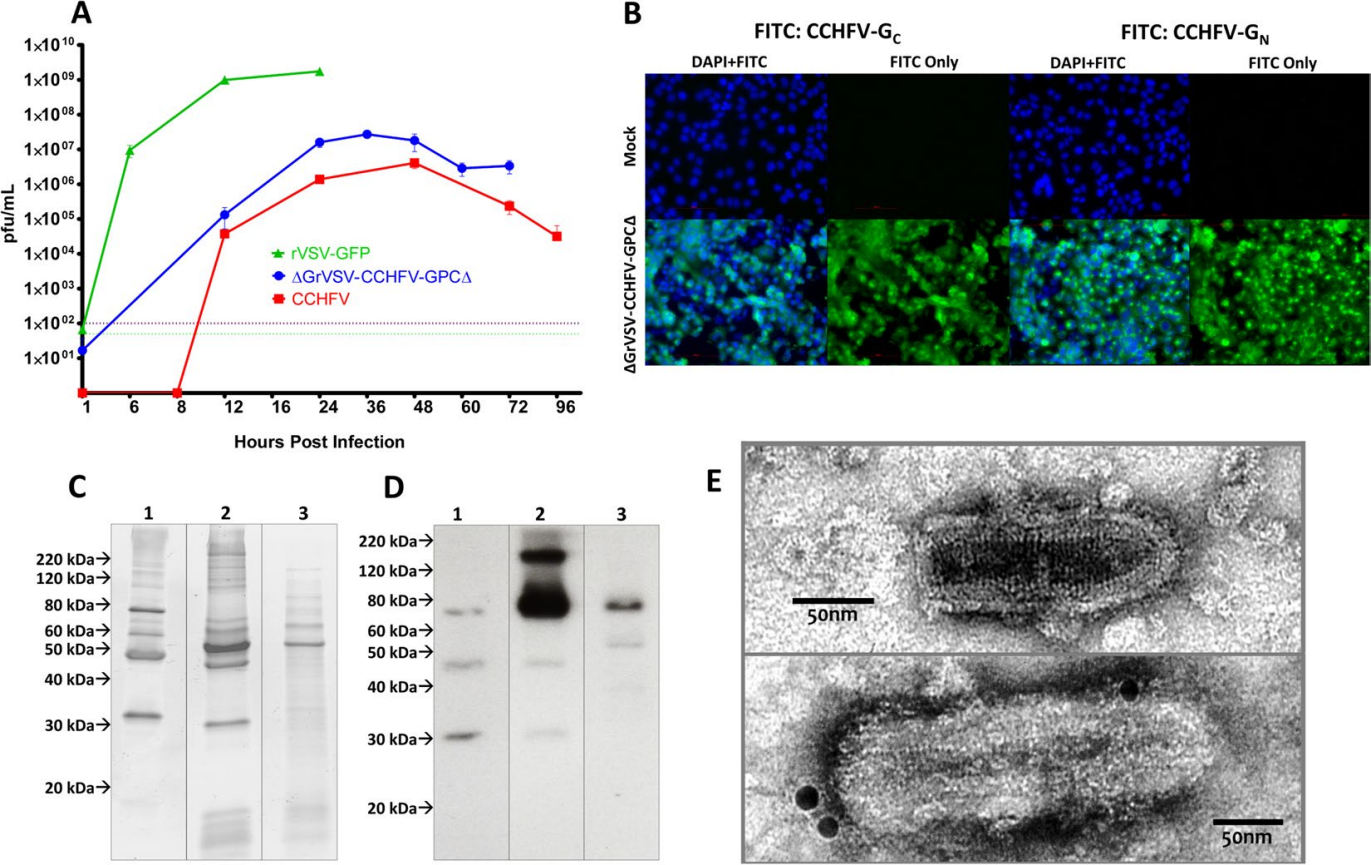
Characterization of rVSV vectors. (**A**) Single-cycle growth kinetics comparing recombinant vesicular stomatitis virus (rVSV) wild-type (Isolate: Indiana) tagged with green-fluorescent protein (GFP) (rVSV-GFP), the replication competent pseudotype (ΔGrVSV-CCHFV-GPCΔ), and Crimean-Congo hemorrhagic fever virus (CCHFV) (Isolate: IbAr10200) all at a multiplicity of infection (MOI 0.1) in baby hamster kidney (BHK) cells. Data shown are a mean ± standard deviation from three biological replicates, titrated by plaque assay in duplicate. (**B**) Fluorescence microscopy of ΔGrVSV-CCHFV-GPCΔ at MOI 0.01 infected human hepatocarcinoma (HuH-7) cells at 20x objective, with a nuclear stain of 4′,6-diamidino-2-phenylindole (DAPI) and GPC antigens stained with α-CCHFV-G_c_ monoclonal antibody (MAb) 8A1 and α-CCHFV-PreG_N_ MAb 13G8 using a Fluorescein isothiocyanate (FITC) conjugated secondary, fixed and permeabilized at 24 hpi, respectively. (**C**) Coomassie of approximately 100 ng of loaded 20% sucrose cushioned/semi-purified and gradient purified particles on 4–16% gradient Mini-PROTEAN TGX (Tris-glycine precast) gels. 1: rVSV-GFP, 2: ΔGrVSV-CCHFV-GPCΔ, and 3: CCHFV. Particle preps were stained with Coomassie Fluor Orange. (**D**) Western blot of approximately 100 ng of loaded 20% sucrose cushioned/semi-purified and iodixanol gradient purified particles on 4–16% gradient TGX gels. 1: rVSV-GFP, 2: ΔGrVSV-CCHFV-GPCΔ, and 3: CCHFV. Particle preparations were stained with α-CCHFV-G_C_ MAb 11E7 using an HRP-conjugated secondary. (**E**) Transmission electron micrographs of rVSV-GFP and replication competent ΔGrVSV-CCHFV-GPCΔ particles from BHK cells, semi-purified using a 20% sucrose cushion or purified using iodixanol gradient centrifugation, negatively stained with 2% aqueous uranyl acetate, immunolabeled with α-CCHFV-G_c_ MAb 11E7 and a secondary 15 nm gold labeled secondary. Samples were fixed with 2% glutaraldehyde. Images were adjusted for brightness, contrast, and formatted for size for display purposes. Coomassie gel and western blot images were cropped from the same image file at same imaging parameters for ease of viewing. Gel and blots originals are available in supplemental information. Other original raw data files available upon request.

To assess the growth kinetics compared to authentic CCHFV, we performed single-cycle growth curve analysis on BHK cells infected with respective viruses at various time intervals up to 96 hrs post infection (hpi) or full monolayer destruction. The rVSV-GFP (wild-type control) peaked in titer at approximately 24 hpi, while CCHFV prototype-strain: IbAr10200 peaked at 48 hpi (Fig. 2A). The replication competent pseudotype, ΔGrVSV-CCHFV-GPCΔ had peak titer, at approximately 36 hpi (Fig. 2A).

To assess the expression of the CCHFV-GPC in the vector, we performed an immunofluorescence assay, using MAbs that bind to either the CCHFV-G_C_ or -G_N_. The assay revealed strong *in vitro* expression of both antigens (Fig. 2B). We additionally examined if CCHFV-G_C_ was incorporated onto the rVSV virion. We used Coomassie staining and western blot analysis on gradient purified ΔGrVSV-CCHFV-GPCΔ and semi-purified rVSV-GFP and CCHFV (as controls). Through Coomassie staining, differences in number and mobility of protein bands were detected among the wt rVSV-GFP and ΔGrVSV-CCHFV-GPCΔ (Fig. 2C, Lanes 1, 2). The CCHFV-GPC recombinant had additional protein bands below 20 kDa with analogous bands detected in CCHFV virion pellets (Fig. 2C, Lanes 2, 3). The CCHFV-GPC recombinant also possessed more pronounced protein bands at 60 kDa, compared to our wt rVSV-GFP (Fig. 2C, Lanes 1, 2). CCHFV structural glycoproteins G_N_ and G_C_ present as bands of approximately at 37 kDa and 75 kDa, respectively (Fig. 2C, Lane 3), on SDS-PAGE gels^7^. There was no observable G_N_ band found on the CCHFV-GPC recombinant at 37 kDa; however, there were two distinct bands between 60–80 kDa in the recombinant (Fig. 2C, Lanes 2). The ΔGrVSV-CCHFV-GPCΔ did not encode for a VSV-G (which was confirmed by our deep sequencing shown in Supplemental Table 1), nor was it complemented with VSV-G after seven rounds of BHK passaging. A duplicate protein gel was run and further probed by western blotting using a MAb previously identified to be specific for CCHFV-G_C_ by western blot (MAb 11E7, BEI Resources)^14^ as there is currently, no available antibody that probes solely mature G_N_ by western blot. These western blot data demonstrated limited, non-specific binding to three VSV proteins as observed in lanes rVSV-GFP (Fig. 2D, Lane 1), but showed strong signal to at least two antigens at approximately 75 kDa and 150 kDa for ΔGrVSV-CCHFV-GPCΔ (Fig. 2D, Lane 2). This Indicated that mature CCHFV-G_C_ and potentially a precursor molecule or an oligomeric form of CCHFV-G_C_ (due to the lack of β-mercaptoethanol in antigen preparations), are incorporated in/on ΔGrVSV-CCHFV-GPCΔ virions. The semi-purified CCHFV also showed limited non-specific binding to antigens at 57 kDa (CCHFV-NP) and 37 kDa (CCHFV-G_N_), but distinct signal at 75 kDa (CCHFV-G_C_) (Fig. 2D, Lane 3).

To examine the ultrastructure of the ΔGrVSV-CCHFV-GPCΔ, transmission electron microscopy studies were conducted. Particles of rVSV-GFP (‘wild-type’ VSV electron microscopy control) were observed between 170–200 nm (Fig. 2E, Top). Consistent with other rVSV pseudotyped with bunyavirus GP^28^, our ΔGrVSV-CCHFV-GPCΔ pseudotype maintained rhabdovirus morphology and classical bullet shape with coiled intra-virion structure (Fig. 2E, Bottom). Particles were observed to have lengths ranging between 210–260 nm (Fig. 2E, Bottom). This apparent length increase of the ΔGrVSV-CCHFV-GPCΔ is likely due to the genome containing approximately 4,000 extra nucleotides in comparison to the rVSV-GFP genome (which is also slightly larger than native wild-type VSV Indiana [GenBank number NC_001560.1]). Additionally, immunolabeling of ΔGrVSV-CCHFV-GPCΔ was employed with MAb to CCHFV-G_C_ and counterstained with 15 nm gold conjugated secondary MAb (Fig. 2E, Bottom). Immunolabeling demonstrated labeling of G_C_ spikes on the virion surface of ΔGrVSV-CCHFV-GPCΔ (Fig. 2E, Bottom).

**Figure 3:**
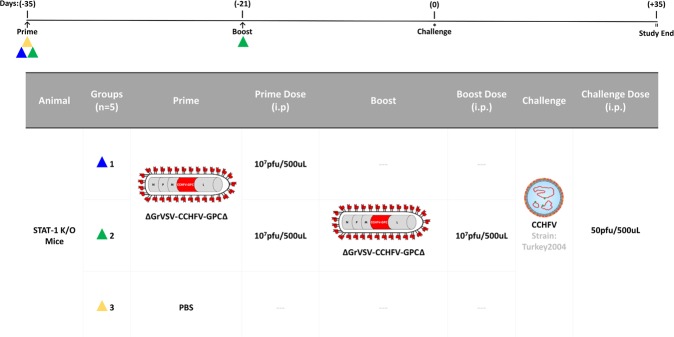
*In vivo* CCHFV vaccine study. Dosing schedule outlined above and experimental conditions including animal type, number, group, prime/boost/challenge conditions, routes, and dose amounts are outlined in the table. Flow chart showing vaccination (triangles), sampling days (arrows), and day of challenge (*).

**Figure 4:**
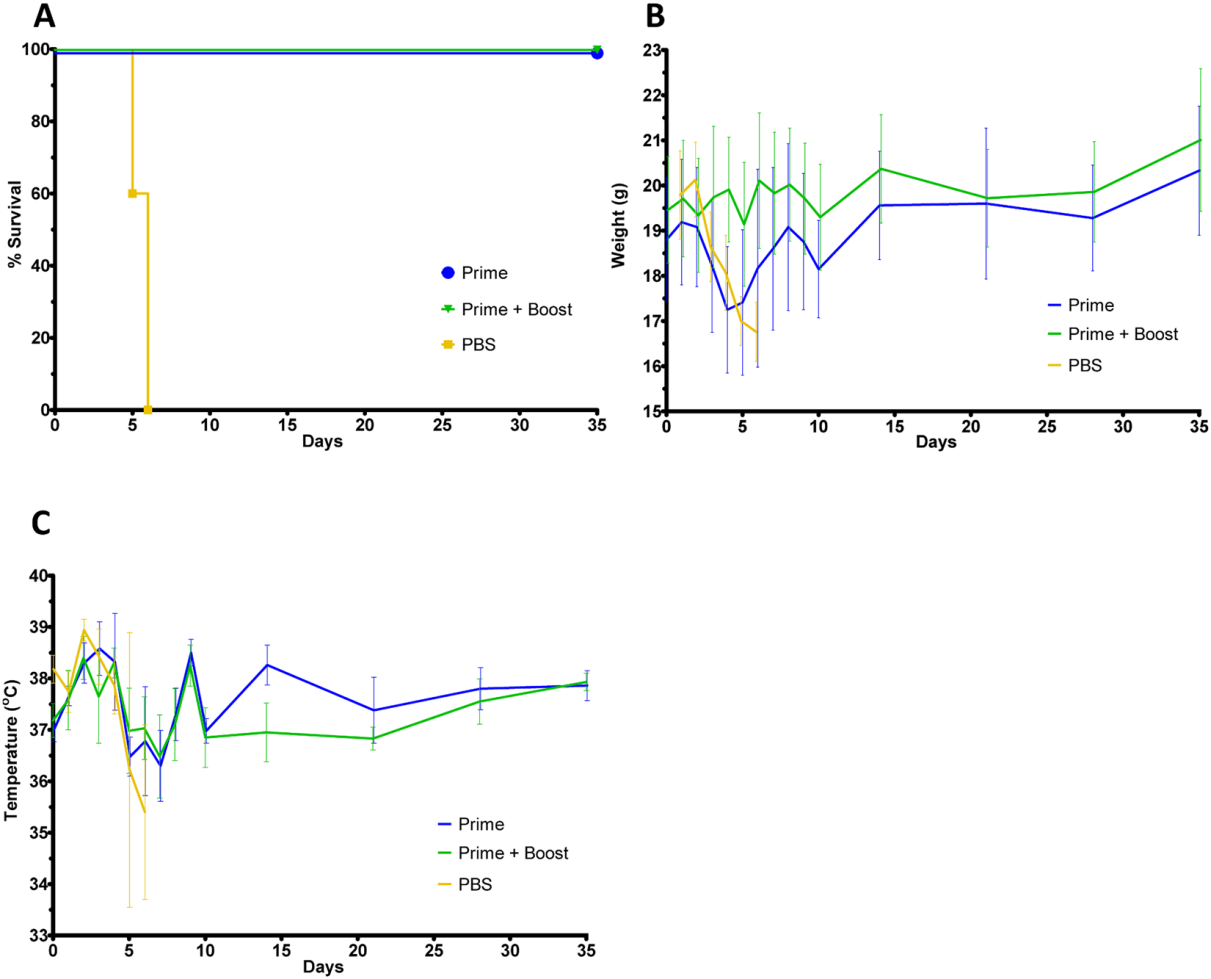
*In vivo* experimental data: animal survival, average weights, average temperatures, and clinical scoring. (**A**) Kaplan-Meier survival curve of challenged animal groups. Group is indicated by color which corresponds to the colored triangles outlined in Fig. 3. (**B**) Averaged weights with error bars from all groups collected each day for 14 days post challenge and every 7 days after that for 35 days post challenge total. (**C**) Averaged temperature with error bars from all groups collected each day for 14 days post challenge and every 7 days after that for 35 days post challenge total.

With the data supporting that the replication competent construct expressed CCHFV-GP *in vitro* and additionally expressed CCHFV-G_C_ on the surface of the virion, an *in vivo* study was designed to test the ability of the construct to function as an experimental vaccine. The STAT-1^−/−^ mouse model for CCHFV was selected to test these constructs for protective efficacy^19^. In pilot studies (Supplemental Fig. 1) the replication deficient construct (VSV-G*-ΔGrVSV-CCHFV-GPC), failed to provide protection; however the replication competent (ΔGrVSV-CCHFV-GPCΔ) construct did demonstrate some protection depending on dose. Based on the results of these initial pilot studies, we elected to adjust vaccine and challenge doses, and administered 10^7^ pfu/dose of the replication competent virus (ΔGrVSV-CCHFV-GPCΔ) to prime and boosted groups of five STAT-1^−/−^ mice, respectively (Fig. 3). As a control, we used PBS as a mock vaccination. A single mouse succumbed in the boosted group (leaving only four mice to be challenged in the boosted group) the day of boosting. At 35 days post prime, all mice were challenged with 50 pfu of CCHFV strain Turkey2004 and were monitored for clinical signs, weights, and temperatures. We challenged with a human/clinical strain, Turkey200406546, designated throughout this work as Turkey2004; which has previously been published in GenBank with the accession numbers KY362517 (S-segment), KY362519 (M-segment), and KY362515 (L-segment)^41^. Mean time-to-death (MTD) was 5.6 days post infection (dpi), with a standard deviation (SD) +/− 0.55 dpi as demonstrated with the PBS control group (Fig. 4A). For both prime and boosted groups, 100% protection was observed out to 35 dpi (Fig. 4A). The prime and PBS group displayed weight loss, while the boosted group did not show weight loss (Fig. 4B). All three groups displayed elevated temperatures for the first three days post challenge (Fig. 4C). These results indicate that the primed group showed a marked illness, while the boosted group did not display observable disease.

**Figure 5:**
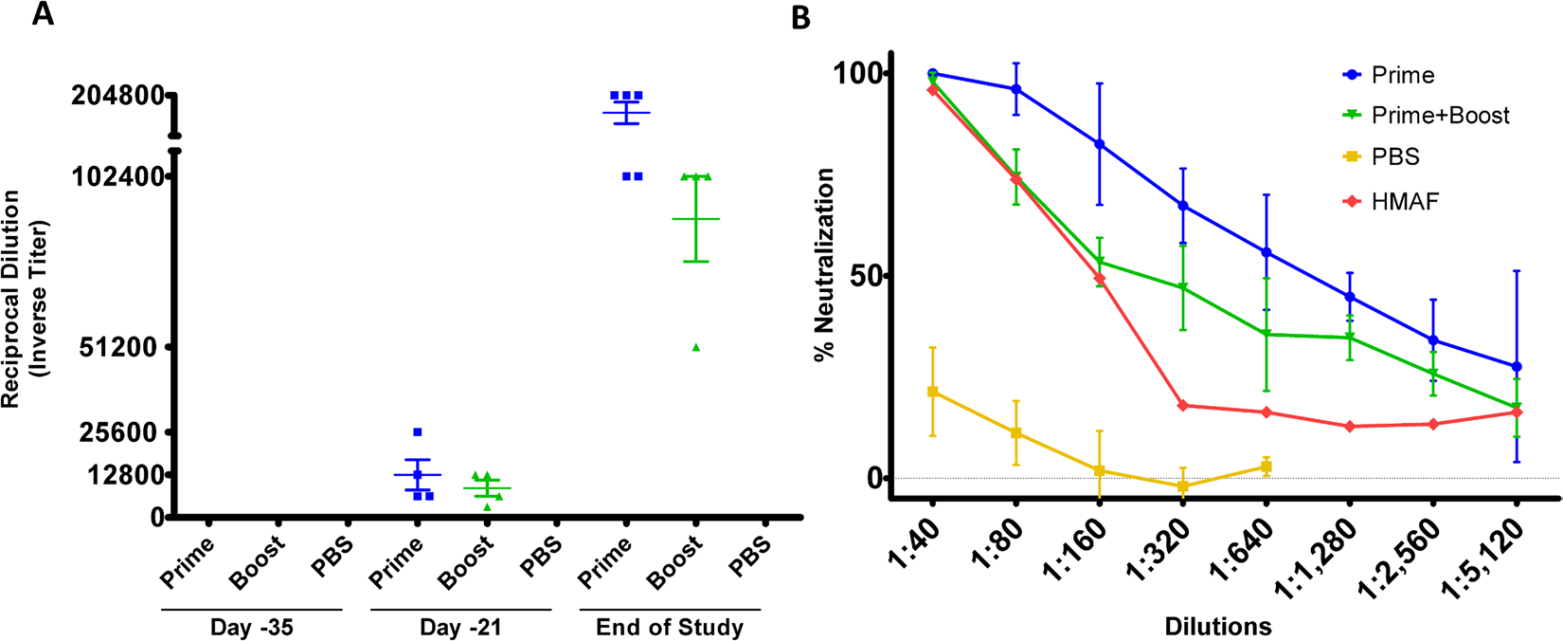
*In vivo* experiment humoral immune responses. Study endpoint mouse sera collected from *in vivo* experiment outlined in Fig. 3 along with pilot study mice from similar doses from pre-challenged status (for days −35 and −21). Mice sera were run in duplicate with each group indicating four separate animal sera tested for humoral responses. (**A**) Enzyme linked immunosorbent assay (ELISA) titers using plates coated with 1ug of purified ΔGrVSV-CCHFV-GPCΔ antigens and diluted various animal sera. (**B**) Plaque reduction neutralization test (PRNT) from diluted endpoint mouse sera with CCHFV (Strain: Turkey2004) incubated on SW-13-CDC cells. Hyperimmune mouse ascitic fluid (HMAF) was used as a positive control, kindly provided by T. Ksiazek.

To examine the humoral response in vaccinated mice, sera from these groups were analyzed for both IgG to CCHFV-GP and for the presence of CCHFV neutralizing antibodies. A separate group of animals was vaccinated (n = 5, per group) at similar prime/boost doses and bled at days −35 and −21 to assess pre-challenged immune status. Circulating IgGs to CCHFV-GP were detected in sera of both vaccinated groups, beginning at day −21 and were found to have increased substantially at the end of study (Fig. 5A). Curiously, at the study end point, the prime group displayed higher titers compared to the prime plus boosted group (Fig. 5A). This indicates the prime only group had a higher concentration of IgG at the study endpoint which recognized CCHFV-GP, compared to the boosted group. The reciprocal titer of the PBS group had negligible IgG responses to CCHFV-GP (Fig. 5A). To determine the neutralizing activity of these sera, a plaque reduction neutralization test (PRNT) was carried out using the Turkey2004 challenge isolate. As depicted in Fig. 5B, the prime only group had neutralizing antibody titers with a PRNT_50_ of < 1:1,280 and the boosted group a PRNT_50_ of < 1:320 at the study endpoint (Fig. 5B). Our PRNT positive control, hyper-immune mouse ascitic fluid (HMAF, kindly provided by T. Ksiazek, Galveston, Texas) raised against CCHFV exposed mice, showed a PRNT_50_ of < 1:160 while the PBS control mice did not demonstrate a PRNT_50_ value (Fig. 5B).

**Figure 6:**
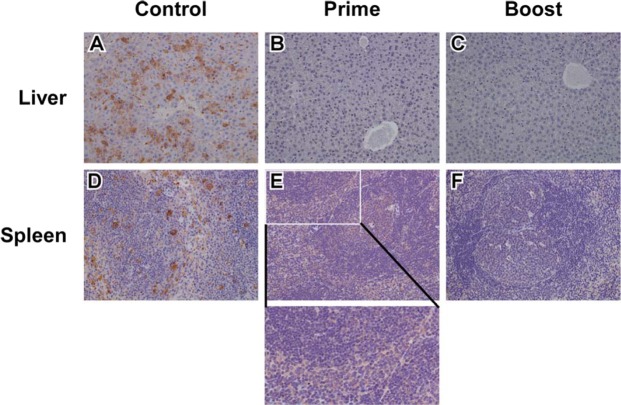
Immunohistochemistry of mice tissues. Representative study endpoint liver tissue sections for the control (**A**), prime (**B**), and boost (**C**) cohorts with observable marked CCHFV immunolabeling of hepatocytes in the control group (**A**) and no observable labeling in the prime (**B**) or boost (**C**) cohorts. Representative study endpoint spleen tissue sections for the control (**D**), prime (**E**), and boost (**F**) cohorts with observable marked CCHFV immunolabeling in mononuclear cells (**D**), cytoplasmic, mild, and diffuse immunolabeling of mononuclear cells primarily in the red pulp (**E**, inset), and no specific CCHFV immunolabeling in the boost cohort (**F**). Original magnification of all panels, 20X.

Additionally, at study endpoints for each cohort, we examined tissues by immunohistochemistry (IHC) for the CCHFV-NP antigen. The control cohort had marked CCHFV-NP immunolabeling in hepatocytes within the liver sections (Fig. 6A) while liver sections from the prime only and boosted cohorts had no observable CCHFV-NP immunolabeling (Fig. 6A,B). We further examined the spleen tissue sections and observed that the control cohort had marked CCHFV immunolabeling in mononuclear cells (Fig. 6D), and that the prime cohort had a cytoplasmic, mild, and diffuse immunolabeling of mononuclear cells primarily in the red pulp (Fig. 6E). The boosted cohort had no specific CCHFV immunolabeling within the spleen sections (Fig. 6F).

## Discussion

Studies have had varied success expressing CCHFV-GPC *in trans* on viral vectors^42,43^. rVSV with CCHFV-GPC has likely been challenging due to the numerous post-translational modifications required to mature, and yield functional, CCHFV-GPC^7,8,10,12^. Additionally, the CCHFV-GPC can form immature CCHFV particles at the Golgi and egress via vesicular transport^14,44^. Unlike CCHFV, VSV buds from the plasma membrane^45^, which hampers efforts to recover a replicating ΔGrVSV vector with CCHFV-GPC expressed. We used a mammalian codon optimized CCHFV-GPC which has demonstrated expression of CCHFV-G_C_ to the cell surface^20^, while maintaining native CCHFV-GPC maturation factors. We hypothesized that this expression due to codon optimization could facilitate shuttling of CCHFV-GP to the plasma membrane where VSV could acquire and bud with functional components of the GPC in the viral envelope. Although we were able to drive strong expression CCHFV-GPC *in vitro*, we were unable to generate a replication competent pseudotype virus without initially using VSV-G* *in trans*. There have been multiple studies that have examined pseudotyping rVSV with a CCHFV-GPC using a plasmid encoding a VSVΔG backbone. A luciferase reporter pseudotype with *in trans* expression of the CCHFV-GPC was developed in 293 T cells by Shtanko *et al*.^43^. Suda *et al*., also performed similar pseudotyping with *in trans* expression in 293 T cells with various full length GPC and modified GPC constructs containing truncated G_C_ C-terminal/endodomain tails^42^. These pseudotypes also had a luciferase or GFP ORFs genomically encoded in the VSVΔG backbone^42^. Both published pseudotyped CCHFV-GPC systems were used for neutralization or entry/infection studies; however, these constructs were not characterized by western blot or immune electron microscopy to understand what is on the pseudotype envelope; nor were either constructs self-replicating or capable of further expressing antigen elements of CCHFV-GPC post infection^42,43^.

With the tools currently available, we were able to detect by western blot and electron microscopy, a form of CCHFV-G_C_ that was present and functional on the surface of the ΔGrVSV-CCHFV-GPCΔ virion. The current situation with reagents to mature CCHFV-G_N_ limit assaying for this particular antigen, as commercially available antibodies only target the precursor molecule CCHFV-PreG_N_. Immune electron microscopy demonstrated that CCHFV-G_C_ was incorporated on the surface of the virion. While we were able to immunolabel for CCHFV-G_C_, PAGE and Coomassie staining analysis of our purified virion lysates did not reveal prominent protein bands at the 37 kDa position, the estimated size of mature G_N_^7^. Nonetheless, we did have three smaller protein bands in the ΔGrVSV-CCHFV-GPCΔ preparations. Similar protein profiles have been shown in other vectored CCHFV-GPC preparations, as shown by Buttigieg *et al*., 2014. It is possible that these protein bands may also correspond to VSV-M2 and -M3 by alternative initiation at downstream start codons present in the ORF of VSV-M^46^. While not the focus of this current study, these observations warrant further characterization should this vector be used as a tool to further study CCHFV-GPC mechanisms.

Suda *et al*., have shown an increase in the amount of infectious pseudotype the more the CCHFV-G_C_ tail is truncated, up to a deletion of 53 residues at the end of the C-terminal CCHFV-G_C_ tail^42^. Our data supports this region as a probable, or at the very least, contributory mechanism which enables the replication competent ΔGrVSV-CCHFV-GPCΔ pseudotype formation, as our pseudotype also was found to contain a C-terminal CCHFV-G_C_ tail truncation (Fig. 1B). There is likely a localization or retention signal within the transmembrane region or endodomain tail which is aberrated and fails to transport CCHFV-G_C_ solely to the intracellular compartments; this may in turn permit shuttling to the plasma membrane. Once again this is not the focus of this study, although it will be examined in the future. It is interesting to note, that a similar motif has been demonstrated by other bunyaviruses^47,48^ and has also been demonstrated with other VSV pseudotypes^49^. Structural CCHFV-G_C_ has been shown to be an important protein for CCHFV and contains a putative receptor binding region for mammalian cell surface nucleolin^50^, a class II viral fusion domain^51^, a neutralization epitope^13^, and human linear B-cell epitope sites^52,53^. Our replication competent rVSV vector can enable further exploration of these components at lower containment (BSL-2), without the need for transfections for *in trans* expression of CCHFV-GPC onto VSVΔG systems.

When exploring the use of rVSV expressing CCHFV-GP as potential vaccines in immunocompromised mice, there was a concern of murine virulence, which has been observed for wild-type VSV^54,55^. Studies with rVSV expressing hemorrhagic fever virus GP have demonstrated lethal outcomes in the STAT-1^−/−^ model^56^. This has hampered the STAT-1^−/−^ animal platform from serving as a vaccine development tool, as ‘vaccinated’ mice succumb to a prime dosing^56^. Because of this information we conducted several pilot studies examining effective dosing and challenge strategies, for both the replication deficient (VSV-G*-ΔGrVSV-CCHFV-GPC) and replication competent (ΔGrVSV-CCHFV-GPCΔ) construct. Demonstrating the safety profile of both constructs (Supplemental Fig. 1), neither group had attributed fatality due to the vaccination; however, only our replication competent construct demonstrated protection. In selecting the CCHFV challenge isolate, we chose an isolate with low passage history and demonstrated clinical relevance (*i*.*e*., documented history of disease in humans). The Turkey2004 isolate, came from a clinical case^41^.

ELISA and PRNT experiments on study endpoint sera, demonstrate a humoral IgG response to CCHFV-GPC with observed neutralizing antibodies produced from the prime group that received a high (10^7^ pfu) dose of ΔGrVSV-CCHFV-GPCΔ (Fig. 5A,B). Curiously, our boosted (10^7^ pfu) group had lower detectable antibodies to the CCHFV-GPC with lower neutralization titers (Fig. 5A,B). Clinical data for these groups following CCHFV challenge show that the prime only group had elevated temperatures and weight loss, additionally two animals from this cohort displayed clinical signs including roughed fur indicating illness from the CCHFV challenge (Fig. 4B,C, data not shown). While the boosted group did display elevated temperatures, there was neither substantial weight loss detected nor observable changes in clinical scoring for any of the animals (Fig. 5B,C, and *data not shown*). Further analysis of tissues at study end point for these cohorts also revealed that while there was no immunolabeling in the liver of the vaccinated animals, the prime cohort had diffuse cytoplasmic immunolabeling of mononuclear cells in the spleen sections (Fig. 6E). This finding, along with the clinical scores, suggested that there was more replication of CCHFV in the prime group compared to the boosted group. This could potentially lead to the higher anti-CCHFV-GPC IgG titers in the prime cohort at the study endpoint as the CCHFV challenge may have served as a heterologous booster, increasing levels when compared to the ΔGrVSV-CCHFV-GPCΔ boosted cohort (Fig. 5A,B). Regardless, protection was achieved by both regimens, although the boosted group data suggests that at study endpoint, the observed IgG titers against CCHFV-GPC along with lower neutralizing titers (PRNT_50_ of < 1:320) are evidence of the ability to combat lethal CCHFV infection in the STAT-1^−/−^ mouse model after vaccination (Fig. 5A,B).

Correlates of protection against CCHF have been difficult to define due to the multiple vaccine and delivery platforms examined to date^16^. Several CCHFV experimental vaccines studies have identified cell-mediated and humoral involvement, with some instances of neutralizing antibody production^24,57^. In looking at what is known for human CCHF, survivors mount a humoral response whereas those who succumb, typically lack an IgG response^3^. Other studies have examined antibody and neutralizing responses from the various vaccine platforms. DNA vaccines following a three round vaccination regimen have induced detectable antibodies with neutralizing capacity observed up to 1:160 in PRNT dilutions and achieved 100% protection, depending on the antigens encoded on the DNA plasmids^25,58^. A cell culture based inactivated virus vaccine achieved high IgG titers (1:102,400) and a high neutralizing response of 1:1,024; however, this also required three vaccination rounds, an alum adjuvant, and conferred 80% protection against the clinical isolate Turkey-Kelkit06 in IFNAR mice^21^. Antibody titers, neutralization capacity, and challenge virus presented here were similar, though through the VSV platform, we achieved greater protection with a single dose. These studies, along with our own, suggest other facets of the immune system are likely involved in conferring complete protection.

As this study was a proof of concept study and not a correlate study, T cell responses were not evaluated, and could be contributory as other groups have shown^23,25,26^. Now that we have established that this new vector can provide protective benefit, our future studies will temporally examine the antibody and T cell repertoire after prime and boosting doses following ΔGrVSV-CCHFV-GPCΔ, but before CCHFV challenge, as these would be informative for the STAT-1^−/−^ mouse model. In the future, the use of the IS murine model might provide additional insights using an intact murine immune systems for vaccinations with a lethal model for CCHFV infections^20^. Ideally, an immunocompetent, larger animal model is greatly needed in the CCHF field to further test the array of CCHFV experimental vaccines which have shown promise in these mouse models. A promising step forward appears to be the nonhuman primate disease model that has been recently developed^59^. However, CCHF in this model appears strain and challenge route dependent.

In conclusion, this study offers not only a tool to study the biology off CCHFV as it relates to structural G_C_, but also serves to develop and characterize a replication competent pseudotype, in relation to CCHF vaccine development. The replication competent construct provides up to 100% protection with an observed humoral response, with a single-injection from a human isolate challenge strain. This information is valuable in designing future studies in CCHFV animal models, and establishes characterized tools to examine the biology of structural CCHFV-G_C_ in a pseudotyped rVSV system.

## Materials and Methods

### Cell culture, challenge virus, and antibodies

Baby hamster kidney cells (BHK) (kindly provided by M. Whitt, University of Tennessee Health Science Center, Memphis, TN), African green monkey kidney E6 clone cells (Vero E6) (American Type Culture Collection [ATCC], Manassas, VA), a clone from the SW-13 human adrenocortical carcinoma cell line (SW-13-CDC) (kindly provided by É. Bergeron of Centers for Disease Control and Prevention, Atlanta, GA), and human hepatocarcinoma cells (HuH-7) (purchased from FisherScientific [XenoTech]) were maintained in Dulbecco’s modified Eagle’s medium (DMEM) supplemented with 10% fetal bovine serum (FBS; Invitrogen, Carlsbad, CA), 2mM L-glutamine (Invitrogen), and 1% penicillin-streptomycin (P/S; Invitrogen), cumulatively called D10. CCHFV challenge stocks, strains IbAr10200 and Turkey-200406546 [referred as Turkey2004, throughout] (kindly provided by T. Ksiazek, UTMB – World Reference Center for Emerging Viruses and Arboviruses, Galveston, TX), were propagated in Vero E6 cells once, plus previous passages in suckling mice and Vero cells since isolation. All *in vitro* and *in vivo* work with CCHFV was performed in a biosafety level 4 facility at the Galveston National Laboratory, University of Texas Medical Branch, Galveston, TX. All cell and viral stocks were tested and free of mycoplasma, by PCR kit (IntronBio, Gyungg-Do, South Korea). Monoclonal antibodies (MAb) mouse-α-CCHFV-G_C_ 11E7 and 8A1, and mouse-α-CCHFV-PreG_N_ antibody 13G8 were generated and characterized as described previously^13,14^. Described antibodies are available at BEI Resources (ATCC) except for 8A1, which was kindly provided by United States Armed Forces Research Institute for Infectious Diseases, Frederick, MD.

### Generation of ΔGrVSV vectors

The rVSV vector was cloned and recovered from cDNA as previously described^60^. Briefly, a BlueScript backbone plasmid under T7 polymerase promoter control encoding a ΔG, VSV Indiana backbone expressing a chimeric *Zaire ebolavirus* (ChEBOV) glycoprotein (GP), was used as the vector (designated pVSVΔG-ChEBOV-GP-3). This plasmid was modified by cutting out an existing ChEBOV-GP coding sequence via *MluI* and *NheI* restriction sites, yielding a pVSVΔG vector. An insert, coding for the codon optimized open reading frame of the complete CCHFV glycoprotein precursor (GPC), was digested and ligated into the pVSVΔG vector. The CCHFV-GPC insert was created by overhang PCR mutagenesis; flanking a 3′ *MluI* restriction site plus Kozak sequence, and a 5′ *XbaI* restriction site, from a PCR amplified, codon optimized, pCAGG-CCHFV-GPC (kindly provided by J. Kortekaas, Central Veterinary Institute, Lelystad, Netherlands). This ligated and cloned plasmid, designated pVSVΔG-CCHFV-GPC, was transfected into BHK cells that were also co-transfected with VSV protein N, P, G, and, L ‘helper’ plasmids under T7 promoter control and driven by infection (MOI 5) with recombinant vaccinia virus expressing T7 polymerase (rVV-TF7-3; kindly provided by M. Whitt). Recovered virus, designated VSV-G*-ΔGrVSV-CCHFV-GPC, was collected 24–48 hrs post infection/transfection, filtered twice through a 0.2 μm filter to remove vaccinia virus, plaque purified onto VSV-G* complemented BHK cells, and stored at −80 °C for further use. All plasmid maps and cloning primer sequences are available upon request.

### Infections, enumeration, growth kinetics, and preparation of viral material

For infections of replication deficient vector, semi-confluent (~60–80%) monolayers of BHK cells were transfected (Lipofectamine LTX, Invitrogen) with 1 μg/1 × 10^6^ cells using pCAGG-VSV-G_(Indiana)_. Once monolayers displayed cell rounding and syncytia, they were infected at an MOI of 0.1 with VSV-G*-ΔGrVSV-CCHFV-GPC. Supernatants were harvested at 24 hrs post infection (hpi) and clarified at 2,000 rpm for 10 min at 4 °C. Confluent monolayers of BHK cells were infected with replication competent ΔGrVSV-CCHFV-GPCΔ at MOI 0.1 for 1 hr at 37 °C with 5% CO_2_ with rocking at 15 min intervals and harvested/clarified at 48 hpi. Plaque assays for viral titrations were carried out in an analogous manner, with an overlay media final concentration of 1.25% Avicel RC-581 (FMC BioPolymer, Philadelphia, PA) in 1x Eagles minimum essentials medium (MEM) with 5% FBS and 1% P/S on BHK cells for 24–48 hpi. After incubations, overlays were aspirated and a 10% buffered formalin fix with a 1X crystal violet stain was incubated onto monolayers for one hr. Plaques were enumerated and plaque forming units (pfu) were determined by averaging technical replicates per sample. Single-cycle growth curves at MOI 0.1 were performed by absorbing rVSV-GFP, ΔGrVSV-CCHFV-GPCΔ, or CCHFV onto duplicate monolayers of BHK cells in six-well plates as described above. Inoculum was aspirated, cells were washed three times with PBS, and D5 was added to monolayers and incubated at various time points indicated in Fig. 2A. At designated time points, samples were collected, clarified, and stored at −80 °C until titrations were performed described above.

### CCHFV-G_C_ protein analysis

Immunofluorescence analysis was carried out by infecting HuH-7 monolayers at a MOI of 0.1 for 24 hpi. Cells were fixed with 4% (w/v) paraformaldehyde, permeabilized with 1% Triton X-100 and stained with 1:500 diluted MAb 8A1 or 13G8. Cells were washed, blocked with 5% BSA, and incubated with a dilution of 1:1,000 secondary goat-α-mouse MAb conjugated to fluorescein isothiocyanate (FITC)(Invitrogen). Cells were mounted and counterstained with the nuclear stain of 4′,6-diamidino-2-phenylindole (DAPI) (Invitrogen). Stained cells were examined on a Nikon Eclipse Ti-S fluorescence microscope. Whole virions were analyzed by infecting BHK cell monolayers, semi-purifying clarified supernatants over a 20% sucrose cushion at 37,000 rpm for 45 min at 4 °C using a Beckman SW 41 Ti rotor. Viral pellets were lysed using NP-40 with 1x Protease Inhibitor Cocktail (Invitrogen). Recombinant VSV lysates were incubated at 56 °C for 10 min and protein subsequently quantified using BCA Protein Assay per manufacturers’ instructions (Thermo Fisher Scientific, Waltham, MA). Per institutional inactivation protocols, CCHFV lysates had a modified inactivation protocol using instead 2x Laemmli Sample Buffer (LSB) (Thermo Fisher Scientific) at 95 °C for 15 min boiling and transfer to a fresh tube. Approximately 200 ng of purified and semi-purified virion associated total protein was mixed 1:1 with 1x LSB (without β-mercaptoethanol) and run on 4–20% gradient Mini-PROTEAN TGX electrophoresis gels (Bio-Rad, Hercules, California). Coomasie staining was accomplished via incubating TGX gels in Coomassie Fluor Orange protein gel stain (Thermo Fisher Scientific) per manufacturers’ instructions and imaged at 300 nm on a Gel Doc XR+ gel documentation system (BioRad). Western blots were run on TGX gels using wet tank transfer to Hybond-P polyvinylidene difluoride (PVDF) membranes (GE Healthcare, Little Chalfont, UK). Membranes were blocked with 5% BSA overnight at 4 °C in Tris/0.1% Tween 20 (Sigma-Aldrich) followed by incubation with primary MAb 11E7 diluted at 1:1,000, overnight at 4 °C. Secondary horse radish peroxidase (HRP) conjugated goat-α-mouse antibody (Thermo Fisher Scientific) was diluted 1:10,000 and incubated on the membrane for 1 h at room temperature. Detection of HRP was accomplished via Pierce ECL western blotting substrate (Thermo Fisher Scientific), Hyperfilm ECL (GE Healthcare), and Kodak Carestream film with X-OMAT 2000 Processor (Eastman Kodak Company, Rochester, NY).

### RNA purification, cDNA creation, and Sanger sequencing analysis

Clarified viral supernatants were placed in TRIzol LS (Thermo Fisher Scientific) at a ratio of 1:5, mixed, incubated for 10 min at room temperature, and transferred to fresh tubes. RNA was isolated from sample mixtures using Zymo Research Direct-zol RNA min-prep (Zymo Research Corp, Irvine, CA), per manufacturers’ instructions. RNA was quantified using a NanoDrop 8000 (Thermo Fisher Scientific) and approximately 500 ng total RNA was used to create cDNA using the SuperScript III First-Strand Synthesis System (Invitrogen) and a VSV-M, matrix protein gene 3′ forward primer. Sanger sequencing on the cDNA was performed using VSV-M, VSV-L, and CCHFV-GPC (codon optimized) open reading frame primer sets and accomplished by the UTMB Molecular Genomics Core using an ABI Prism 3130XL DNA Sequencer (Applied Biosystems, Foster City, CA). Sequence analysis was performed using Geneious R9 (Biomattes, Auckland, New Zealand) based on consensus and plasmid maps. All cDNA/sequencing primers and consensus/plasmid maps are available upon request.

### Deep sequence analysis of viral RNA genomes

To analyze the stocks of CCHFV or rVSV vaccine vectors used in this study we performed deep sequencing analysis of RNA isolated from these virus stocks. Briefly, viral RNA was isolated from a Trizol LS (Invitrogen)/sample mixture using a Direct-zol RNA mini-prep (Zymo Research), per manufacturer’s instructions. Approximately 150 ng of purified RNA was used to make cDNA using the Ovation RNA-seq. 2.0 kit (NuGen) and this in turn was used for the preparation of the double-stranded DNA library, using Encore Ion Torrent library prep kit. Sequencing was performed by the UTMB Molecular Core on the Ion Torrent using 318-v2 deep sequencing chips. Sequence analysis was performed using DNA Star Seqman NGen software (DNA Star) based on unpaired analysis of 125 base pair overlaps.

### Ultrastructural analysis

Viruses were propagated in multiple T-150 flasks of confluent BHK cells. Viral supernatants were harvested and clarified as described above. Clarified supernatants were concentrated by mixing with buffered 4x polyethylene glycol with incubation for 4 hrs at 4 °C, followed by centrifugation at 6,800xg for 30 mins at 4 °C. Concentrated pellets were re-suspended in PBS with protease inhibitor and overlaid atop Optiprep (Sigma-Aldrich) continuous gradients of 6–48% buffered iodixanol. Viruses were banded by ultracentrifugation at 25,000xg for 15 hrs at 4 °C using a SW 41 Ti rotor. Bands were harvested, washed in PBS, and pelleted at 27,000xg for 1 h at 4 °C using a SW 41 Ti rotor to remove residual iodixanol. Purified viral pellets were re-suspended in PBS and absorbed onto Formvar-carbon coated nickel grids (Electron Microscopy Sciences [EMS], Hatfield, PA) for 10–30 mins, incubated with MAb 11E7 at 1:10 dilutions. Antibodies were absorbed for 30 mins in wet chamber and washed with PBS containing 1% BSA, and incubated with the secondary antibody, goat-α-mouse conjugated to 15 nm colloidal gold particles (EMS), at a dilution of 1:20 for 30 mins. Grids were washed with PBS and 1% BSA, fixed using 2% (w/v) aqueous glutaraldehyde for 10 mins, and finally stained with 2% (w/v) aqueous uranyl acetate. Grids were examined at 60 kV using a Philips CM-100 transmission electron microscope.

### Ethics of care, vaccination, and animal challenge

Animal studies were approved by the UTMB Institutional Animal Care and Use Committee (IACUC). Animal research was carried out in compliance with the Animal Welfare Act and other federally regulated stipulations regarding animals and adherences to the *Guide for the Care and Use of Laboratory Animals*, National Research Council, 2013. The animal facilities where this research was conducted are accredited by the Association for Assessment and Accreditation of Laboratory Animal Care International. This study used female 4 to 8 weeks old 129S6/SvEv-*Stat1*^*tm1Rds*^ mice (STAT-1^−/−^) (Taconic, Germantown, NY). After an acclimatization period in barrier conditions in environmentally enriched sterile housing, mice were anesthetized by isoflurane and implanted with subdermal transponders, which provide coded identifiers and permitted body temperature measurements (Biomedic Data Solutions, Seaford, DE). Vaccine preparations were diluted in Hanks balanced salts medium with 2% FBS, along with PBS for control groups. After anesthesia by isoflurane, 500 ul of each preparation was administered intraperitoneally (i.p.), with five STAT-1^−/−^ mice per experimental group. Clinical scoring, body temperature, and weight, were recorded daily. At 35 days post vaccination (prime), mice were challenged with 500 ul i.p. with either 100 pfu of CCHFV-Ibar10200 or CCHFV-Turkey200406546 (referred to as Turkey2004) (however, 50 pfu calculated back titer of challenge preparation for the Turkey2004 administration and is thus reported as such in Fig. 3), respectively. All challenge doses were frozen for storage and verified by back-titrations by plaque assay on SW-13-CDC cells as outlined above. After challenge, animals were observed for clinical scoring, temperature, and weight change. Upon reaching institutionally approved endpoint score criteria, or study end-point, blood samples were collected into K3EDTA containing collection tubes (Granier, USA) and plasma was separated then frozen at −80 °C for storage and further analysis. Euthanasia criteria was defined as: mouse displays severely hunched posture, inability or reluctance to move, appears weak (staggering when moving around cage), labored breathing, or weight loss of greater than 12% of starting body weight.

### Anti-CCHFV-GPC IgG ELISA development

Iodixanol gradient purified ΔGrVSV-CCHFV-GPCΔ and rVSV-GFP were re-suspended in NP-40 buffer and BCA protein quantified (previously described above) and were used as whole virion antigens in coating Immunosorbent 96-well plates (Thermo Fisher Scientific). Matrices of various antigen, blocking, primary antibodies (hyperimmune mouse ascetic fluid [HMAF kindly provided by T. Ksiazek], 8A1, and 11E7) to CCHFV/CCHFV-GPC, and secondary antibody (MAb HRP-goat-α-mouse) concentrations were used to develop optimal detection conditions for the CCHFV-GPC via ELISA. Per optimizations, one microgram of purified antigen per mL was suspended in sterile filtered sodium bicarb/carbonate buffer (pH 9.6) and allowed to incubate on immunosorbent plates overnight at 4 °C. Plates were washed with PBS containing a concentration 0.1% tween-20 and 0.001% thimerosal. Blocking occurred with 5% milk dissolved in wash buffer, for 2 hrs at room temperature. Sera from STAT-1^−/−^ mice was added 1:100 and diluted 2-fold by pipetting across plates and allowed to incubate for one hr at 37 °C. Plates were washed and a secondary anti-mouse antibody conjugated to HRP was added at 1:5,000 dilution for one hr at 37 °C. ABTS Peroxidase substrate (KPL, SeraCare Life Sciences, Milford, MA) was incubated for 15 mins at room temperature prior to the addition of a 1% SDS stop solution. Plates were read with nine reads per well at 405 nm with a plastic correction factor accounted for from a 490 nm reading per well. Test sera was evaluated using both purified ΔGrVSV-CCHFV-GPCΔ and rVSV-GFP antigen.

### Plaque reduction neutralization assay

Serial dilutions of sera from four mice per treatment group, were aliquoted into cluster tubes with D10 and allowed to incubate with 100 pfu of CCHFV, isolate Turkey 2004, for approximately 2 hrs on ice. Resulting sera plus virus mixture was then overlaid onto 6-well plates of confluent SW-13-CDC cells and absorbed for 1 hr at 37 °C with 5% CO_2_ with rocking at 15 min intervals. Plaque assays were carried out in a manner described above in previous methods section. Resulting plaques were enumerated from virus + sera wells and compared to sera plus media only wells run for each sample, and a percent neutralization was calculated and reported for each dilution. Hyperimmune mouse ascitic fluid [HMAF] raised against CCHFV was additionally serially diluted and run as a positive control.

### Immunohistochemistry of tissues

Tissue sections were deparaffinized and rehydrated through xylene and graded ethanols. Slides went through heat antigen retrieval in a steamer at 95 °C for 20 mins in Sigma Citrate Buffer, pH6.0, 10× (Sigma Aldrich, St. Louis, MO). To block endogenous peroxidase activity, slides were treated with a 3% hydrogen peroxide and rinsed in distilled water. The tissue sections were processed for IHC using the Thermo Autostainer 360 (ThermoFisher, Kalamazoo, MI). Sequential 15 min incubations with avidin D and biotin solutions (Vector, Burlingame, CA) were performed to block endogenous biotin reactivity. Specific anti-CCHFV immunoreactivity was detected using a primary polyclonal rabbit-α-CCHFV-NP antibody (IBT BioServices, Rockville, MD) at a 1:3200 dilution for 60 mins. A secondary biotinylated goat-α-rabbit-IgG (Vector Laboratories, Burlingame, CA) at 1:200 dilution for 30 mins followed by Vector Horseradish Peroxidase Streptavidin, R.T.U (Vector) for 30 mins. Slides were developed with Dako DAB chromagen (Dako, Carpenteria, CA) for 5 mins and counterstained with Harris hematoxylin for 30 seconds. Tissue sections from uninfected mice were used as negative controls.

### Statistical analysis

A Kaplan-Meier survival curve was constructed with GraphPad Prism software (Graphpad Software, Inc., San Diego, CA). A power analysis utilizing parameters that included survival, neutralization capacity, and ELISA antibody titers from previous pilot studies was performed to assess adequate animal numbers for the current study to differentiate between surviving groups versus control groups to obtain a statistical significance of p < 0.05 with at least a 90% probability.

## Supplementary information

Supplemental Information
