# Supplementary material for: Vesicular Stomatitis Virus-Based Vaccine Protects Mice against Crimean-Congo Hemorrhagic Fever
Source: Sci Rep. 2019 May 23;9:7755. doi: 10.1038/s41598-019-44210-6 (PMC6533279; doi:10.1038/s41598-019-44210-6)
Supplement: Supplementary file 1 — Supplemental Information [file 41598_2019_44210_MOESM1_ESM.docx]

**SUPPLEMENTAL INFORMATION**

**Title:** Vesicular Stomatitis Virus-Based Vaccine Protects Mice against Crimean-Congo Hemorrhagic Fever

**Byline:** Serio E. Rodriguez, Robert W. Cross, Karla A. Fenton, Dennis A. Bente, Chad E. Mire, and Thomas W. Geisbert

**
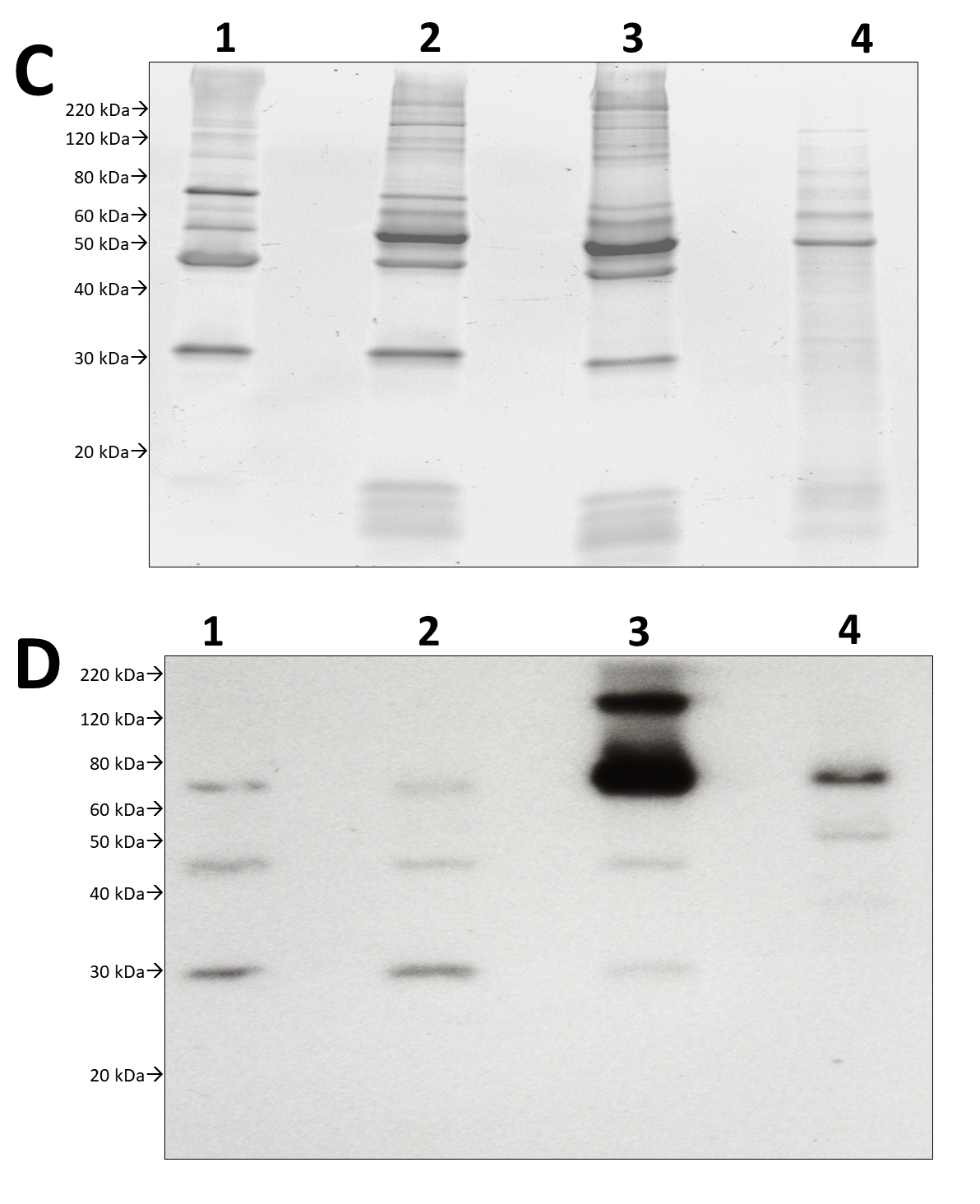
**

**Raw Files for Figures 2C and 2D:** (**C**) Coomassie of approximately 100 ng of loaded 20% sucrose cushioned/semi-purified and gradient purified particles on 4-16% gradient TGX gels. 1: rVSV-GFP, 2: VSV-G*-ΔGrVSV-CCHFV-GPC, 3: ΔGrVSV-CCHFV-GPCΔ, and 4: CCHFV. Particle preps were stained with Coomassie Fluor Orange. (**D**) Western blot of approximately 100 ng of loaded 20% sucrose cushioned/semi-purified and gradient purified particles on 4-16% gradient TGX gels. 1: rVSV-GFP, 2: VSV-G*-ΔGrVSV-CCHFV-GPC, 3: ΔGrVSV-CCHFV-GPCΔ, and 4: CCHFV. Particle preparations were stained with α-CCHFV-G_c_ MAb 11E7 using an HRP-conjugated secondary.


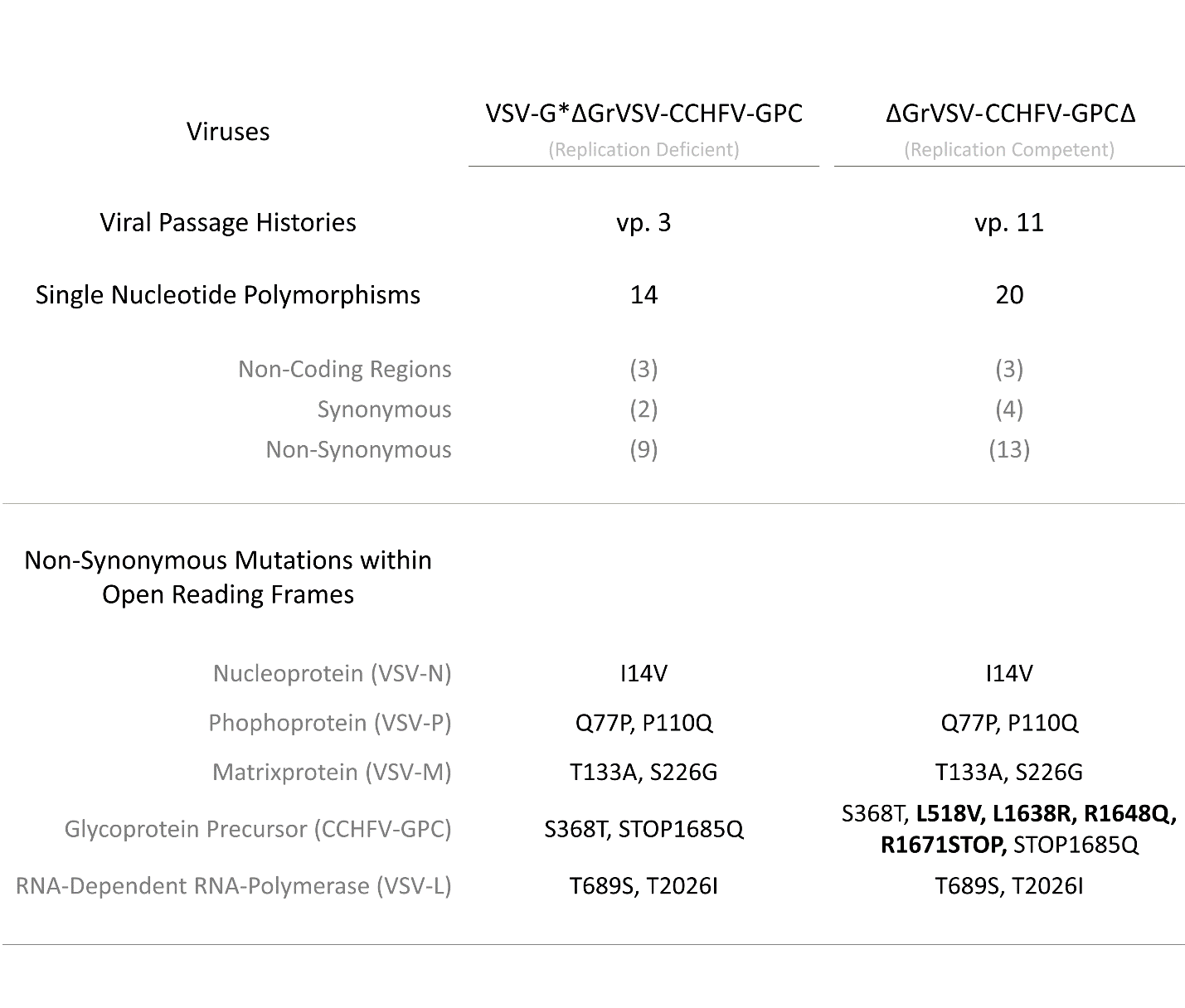


**Supplemental Table 1. Results summary of next generation sequencing of rVSV-CCHFV-GPC Whole Genomes.** Next generation sequencing (NGS) comparisons of single nucleotide polymorphisms (SNP) along with nonsynonymous residue changes between replication deficient VSV-G*-ΔGrVSV-CCHFV-GPC and replication competent ΔGrVSV-CCHFV-GPCΔ pseudotypes. Nonsynonymous residue changes present only in the replication competent pseudotype are bolded.

**
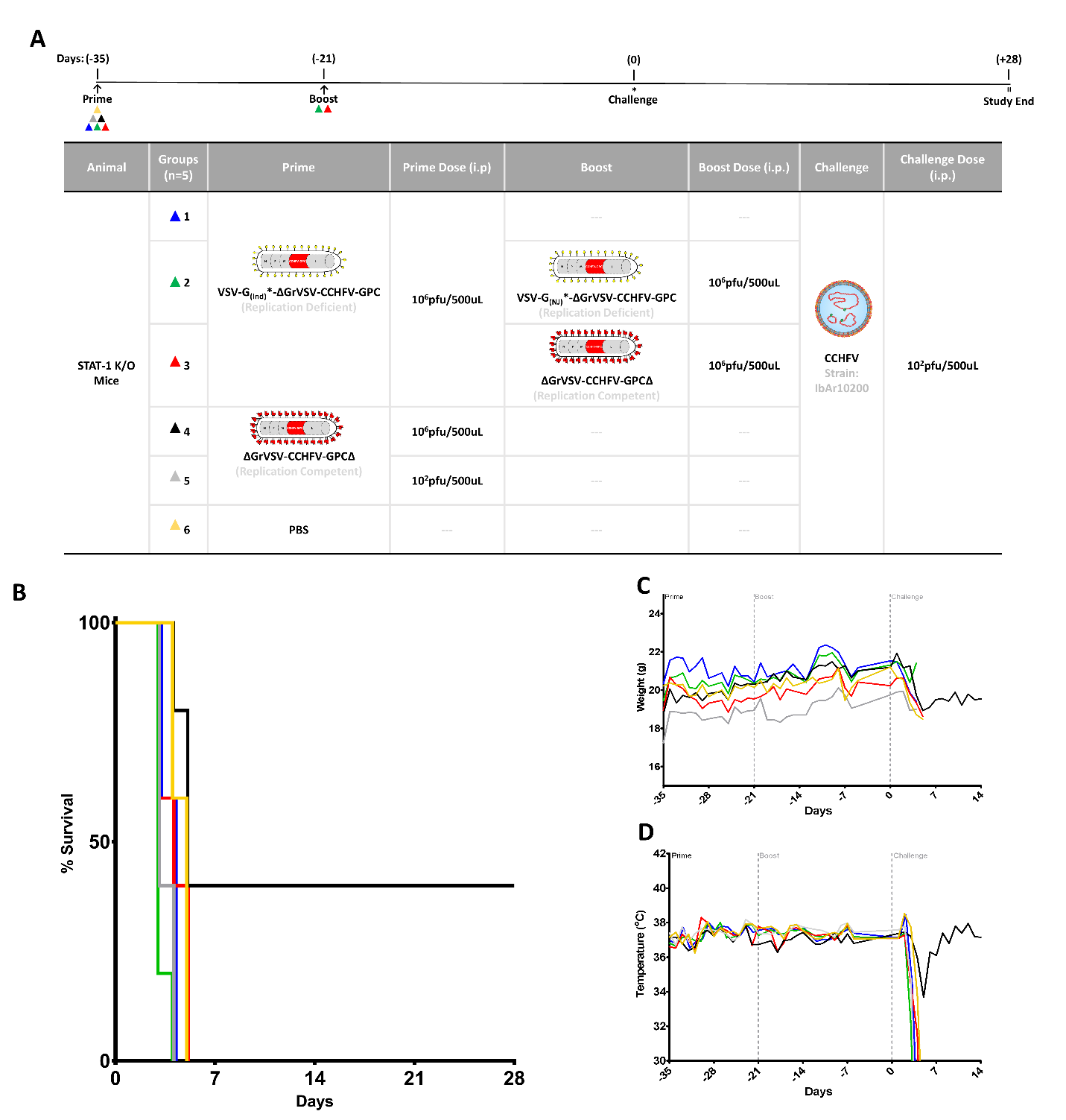
**

**Supplemental Figure 1. Pilot *in vivo* CCHFV vaccine study**. (A) Experimental conditions including animal type, number, group, prime/boost/challenge conditions, routes, dose amounts, and schedule. Flow chart showing vaccination (triangles), sampling days (arrows), and day of challenge (*). (B) Kaplan-Meier survival curve of challenged animal groups. Group is indicated by color which corresponds to the colored triangles outlined in Supplemental Figure 1A, above. (C) Averaged weights from all groups collected each day for 35 days pre-challenge and 14 days post challenge total. (D) Averaged temperatures from all groups collected each day for 35 days pre-challenge and 14 days post challenge total.
